# Bayesian inference of physiologically meaningful parameters from body sway measurements

**DOI:** 10.1038/s41598-017-02372-1

**Published:** 2017-06-19

**Authors:** A. Tietäväinen, M. U. Gutmann, E. Keski-Vakkuri, J. Corander, E. Hæggström

**Affiliations:** 1 Department of Physics, University of Helsinki, FI-00014 Helsinki, Finland; 2 School of Informatics, University of Edinburgh, Edinburgh, EH8 9AB UK; 3 Department of Mathematics and Statistics, University of Helsinki, FI-00014 Helsinki, Finland; 4 Department of Biostatistics, Institute of Basic Medical Sciences, University of Oslo, N-0317 Oslo, Norway

## Abstract

The control of the human body sway by the central nervous system, muscles, and conscious brain is of interest since body sway carries information about the physiological status of a person. Several models have been proposed to describe body sway in an upright standing position, however, due to the statistical intractability of the more realistic models, no formal parameter inference has previously been conducted and the expressive power of such models for real human subjects remains unknown. Using the latest advances in Bayesian statistical inference for intractable models, we fitted a nonlinear control model to posturographic measurements, and we showed that it can accurately predict the sway characteristics of both simulated and real subjects. Our method provides a full statistical characterization of the uncertainty related to all model parameters as quantified by posterior probability density functions, which is useful for comparisons across subjects and test settings. The ability to infer intractable control models from sensor data opens new possibilities for monitoring and predicting body status in health applications.

## Introduction

Upright stance is inherently unstable due to the physics of an inverted pendulum-like body and due to the internal perturbations of an individual, such as noise in afferent (sensory) and efferent (motor) nerve pathways, respiration, and hemodynamics^1–4^. Balance is controlled by co-operating visual, vestibular, and somatosensory systems. The sensory information is integrated in the central nervous system (CNS), which determines the actions needed to maintain balance and which commands the musculoskeletal system to execute corrective actions to maintain an upright stance.

Factors that affect the CNS and skeletal muscles also influence postural steadiness. Therefore, quantifying postural steadiness during upright stance may provide insight into the physiological state of a person. In one kind of posturographic measurement a person stands erect on a force plate while the plate measures the net center-of-pressure (COP) along the mediolateral (ML) and anteriorposterior (AP) directions. The COP signal is closely related to the 2D center-of-mass (COM) signal^5, 6^, the time-varying vertical projection of the 3D body’s center-of-mass. Traditionally COP signals are quantified by statistical sway measures extracted from raw data. These measures typically describe mean sway amplitude, velocity, and frequency^7^. Previously posturographic measurements have been used to quantify effects of aging^7–9^, state of alertness^10–12^, use of anesthetic drugs^13–15^, and conditions, such as multiple sclerosis^16, 17^, and Parkinson’s disease^18, 19^.

Upright stance can be modeled using an inverted pendulum model that depicts the human body as a rigid rod pivoting around its floor-anchored end. The pendulum may have either one or more links that depict human joints, such as ankles, hips, and knees. A single-link (ankle) model is simple and common, and it can be used to describe quiet, upright stance^9, 20, 21^. Modeling large movements due to i.e. perturbations of voluntary movements require two^22^ (ankle and hip) or more links^23, 24^. Due to internal and external disturbances, such as gravitation and respiration, the pendulum body needs to actively be maintained in an upright position to avoid falls. Two types of control exist: the passive controller mimics the effect of tendons and muscle tone that act instantaneously (no delay), whereas the active controller mimics actions taken by the CNS, where a time delay is present. The simplest form of control relies on passive ankle stiffness alone where no active control by CNS is present^25^. However, direct measurements on ankle stiffness have shown that passive stiffness alone can not maintain stable posture^26–28^. More recent models feature PID (proportional-integrative-derivative)^20^, PD (proportional-derivative)^21, 22^, or optimal^29^ active control together with passive control to maintain balance. These controllers act either continuously^20^ or intermittently^21, 22^, that is, only when they are needed. Recently it was shown that a continuous control model may predict physiologically unrealistic parameter values, especially too much noise^4, 21^.

**Figure 1:**
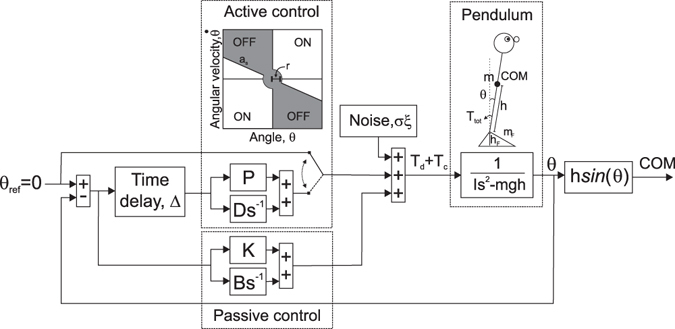
Sway model^21^. Human body is modelled as a rigid, weightless inverted pendulum with a point mass, *m*, at the height, *h*, of the body’s center-of-mass (com), rotating around a single link (ankle). The perturbations or noise, *σ*, due to gravitation and internal and external disturbances, such as blood circulation and respiration, cause the body to be unstable. Passive (stiffness *K*, damping *B*) and active (stiffness *P*, damping *D*) control (here proportional and derivative controllers) calculate a control torque, *T*_*c*_, that is needed to counteract the torques caused by disturbances and gravitation (*T*_*d*_ and *T*_*g*_). Passive control describes continuous muscle tone, and active control –that acts with time delay, *Δ*,– describes CNS action. Active control is ‘ON’ only when needed, according to the magnitude and direction of body angle, *θ*, and angular velocity, $\dot{{\boldsymbol{\theta }}}$. The level of control, *C*_*ON*_, models this amount or fraction of active torqueing. Finally, *s* is the Laplace transform variable. Five parameters were chosen for the inference: *P*, *D*, *Δ*, *σ*, and *C*_*ON*_. Please see explanations for rest of the parameters in Section Methods (The control model).

We focus here on the model presented by Asai *et al*. in 2009 where the body is depicted as a single-link inverted pendulum (SLIPM)^21^. In the Asai model the body is kept upright by an active and a passive PD (proportional, derivative) controller. Whereas the passive controller acts continuously, the active controller acts intermittently. The active control corrects the posture only when necessary, depending on pendulum angle, and angular velocity^21^. The intermittent control employs parameter values that are more physiologically plausible than those of earlier models^21^. The model is described in Fig. 1 and in Section Methods (The control model).

In a realistic characterization of human sway behaviour, the model parameters serve as biomarkers which can be interpreted physiologically. In earlier work, Maurer and Peterka measured COP signals and modeled them using SLIP model with continuous control^20^. The authors showed that the control parameters differed between young and elderly. However, neither their study nor other related studies did consider formal statistical inference since the model properties render likelihood calculations intractable. Consequently, formal statistical quantification of model parameters and predictive uncertainty has, to our knowledge, not previously been considered in this context.

Bayesian inference provides a principled framework to deduct posterior probabilities of the model parameters from the measured data. The likelihood function is a key ingredient for the calculation of the posterior probabilities. However, it is quite common that the likelihood function is unavailable analytically in closed form and that accurate numerical approximations are computationally too expensive as well. Markov Chain Monte Carlo (MCMC) methods have been developed to address this issue and they have been successfully applied, among others, to genetics^13–15^, infectious disease epidemiology^30, 31^, and climate research^32, 33^. However, MCMC methods have their limitations and for complex models they “too inefficient by far”^34^. Approximate Bayesian computation (ABC) is an alternative inference technique that can be used when other techniques are not applicable. It is approximate since it operates on summary statistics of the data rather than the raw data themselves. While approximate, it has been shown to produce accurate approximations of the posterior probability distribution as compared to those produced by using exact inference methods for tractable models^34–36^. ABC has rapidly gained attention in many of the same application fields as MCMC, such population genetics^37^ and infectious disease epidemiology^38, 39^, and we use it in this paper for posterior inference. In particular, we show that approximate Bayesian computation together with the SLIP model can accurately infer sway characteristics of both simulated and real test subjects.

## Results

**Figure 2:**
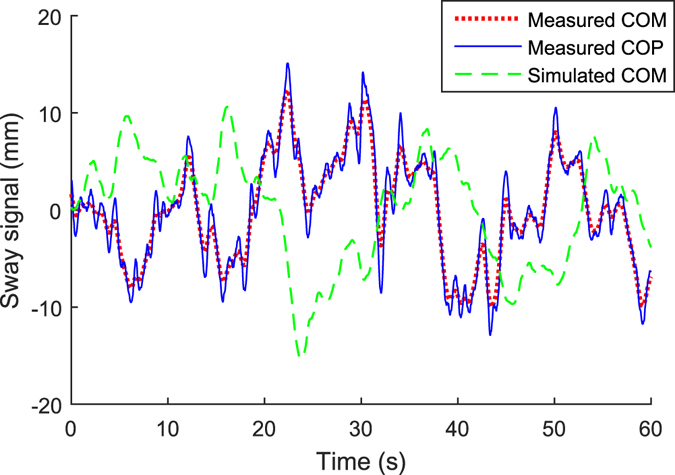
Manifestation of measured COP and COM signals, and of a simulated COM signal. The measured COM is calculated from the COP signal using Eq. (9).

Figure 1 presents the schematic of the Asai 2009 sway model^21^ that outputs COM signals. Section Methods (The control model) presents the details of the model. In this study, we focus on the following five parameters of interest: Active stiffness (*P*), active damping (*D*), time delay (*Δ*), noise (*σ*), and level of control (*C*_*ON*_). These model parameters were inferred as described in the Section Methods (Statistical inference of the model parameters). Figure 2 shows a COM signal generated by the model and an example of a measured COP signal together with its COM signal, computed according to Eq. (9). The measured COM signal follows the general trend of the COP signal, but is smoother.

The main results are presented in the following two sections. Section Simulated subjects presents examples of simulated and inferred COM signal and summary statistics, examples of marginal posterior probability density functions (PDFs) of the parameters of interest, the overall accuracy of the inferences, and finally the sensitivity analysis. Section Real subjects presents the same results as Section Simulated subjects but with real subjects. In Section Real subjects the level of accuracy of the inferences is quantified by comparing sway measures calculated from the original and inferred COM signals, since the true parameter values are unknown.

### Simulated subjects

This section demonstrates that the ABC inference algorithm accurately infers the parameters of interest from the Asai 2009 model^21^ output, using the method described in Section Methods (Statistical inference of the model parameters). For this, we created 10 simulated subjects that are described in detail in Section Methods (Test subjects and measurements).

**Figure 3:**
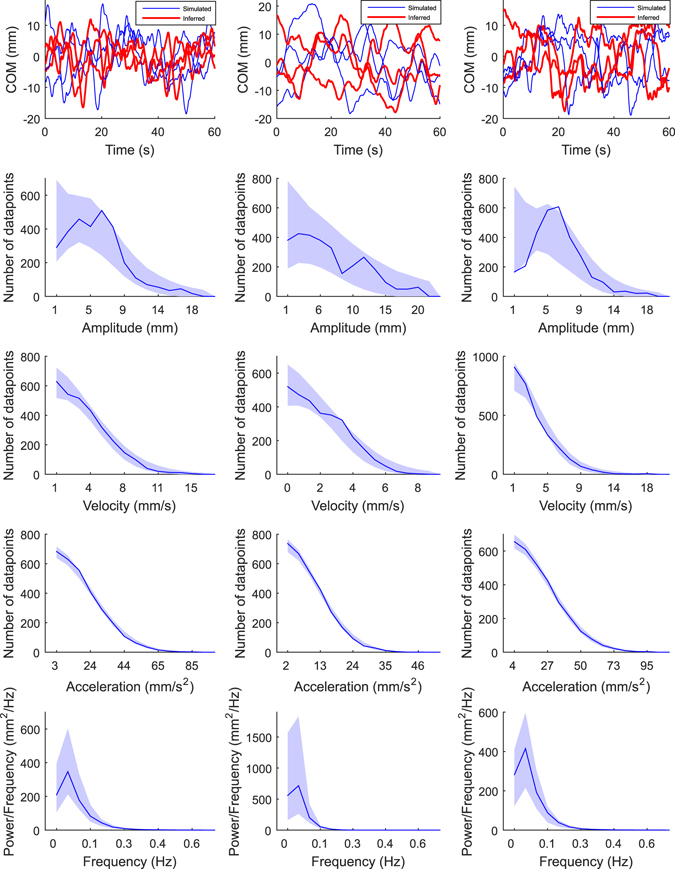
Simulated COM sway signals (top panel) and corresponding summary statistics (four lowest panels). The three columns present three simulated test subjects. The blue COM signals are the original three signals that were simulated with known parameter values. The red COM signals were simulated using parameters that were sampled from the joint posterior PDFs that were inferred from the original COM signals by the SMC-ABC algorithm (see Section Methods: Statistical inference of the model parameters). The lower panels show the summary statistics: amplitude -, velocity -, and acceleration histograms and spectra. In each panel, the blue line is the true summary statistic calculated from the original COM signals (average of the three), and the blue shadowed region presents 95% CIs that were calculated from the COM signals simulated using parameters that were sampled from the inferred marginal posterior PDFs. Leftmost column: true parameters: *m* = 54 kg, *h* = 0.85 m, *P* = 124 Nm/rad, *D* = 8.7 Nms/rad, *Δ* = 0.16 s, *σ* = 0.22 Nm, *C*_*ON*_ = 0.63; estimated parameters: *P* = 119 Nm/rad, *D* = 24 Nms/rad, *Δ* = 0.18 s, *σ* = 0.23 Nm, *C*_*ON*_ = 0.67. Mid column: true parameters: *m* = 80 kg, *h* = 0.78 m, *P* = 128 Nm/rad, *D* = 37 Nms/rad, *Δ* = 0.16 s, *σ* = 0.16 Nm, *C*_*ON*_ = 0.75; estimated parameters: *P* = 126 Nm/rad, *D* = 37 Nms/rad, *Δ* = 0.14 s, *σ* = 0.16 Nm, *C*_*ON*_ = 0.82. Rightmost column: true parameters: *m* = 82 kg, *h* = 0.96 m, *P* = 237 Nm/rad, *D* = 29 Nms/rad, *Δ* = 0.16 s, *σ* = 0.44 Nm, *C*_*ON*_ = 0.59; estimated parameters: *P* = 244 Nm/rad, *D* = 22 Nms/rad, *Δ* = 0.12 s, *σ* = 0.45 Nm, *C*_*ON*_ = 0.59.

Figure 3 presents COM signals from three simulated test subjects. The COM signals were generated with different parameter values (“original” COM signals), and with the corresponding parameter values that were inferred with SMC-ABC algorithm from the original COM signals (“inferred” COM signals). The inferred COM signals are difficult to distinguish from the original COM signals by eye. Lower panels in Fig. 3 present the summary statistics (amplitude, velocity, and acceleration histograms and spectrum) that were used to compare the original COM signals and the inferred COM signals. Figure 3 shows that the summary statistics calculated from the original simulated COM signals fit into the 95% CI area of the summary statistics which describe the COM signals that were simulated using the inferred parameters.

**Figure 4:**
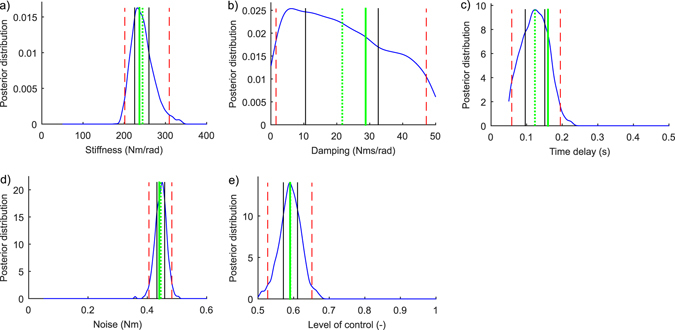
Marginal posterior probability density functions of the five parameters: (**a**) Stiffness, *P*; (**b**) Damping, *D*; (**c**) Time delay, *Δ*; (**d**) Noise, *σ*; and (**e**) Level of control, *C*_*ON*_. Vertical lines present true parameter values (green, thick), estimated parameter values (green, dotted), 50% CIs (black, solid), and 95% CIs (red, dashed). These results are from the same simulated test subject as in the rightmost panel in Fig. 3. The ranges on the x-axes correspond to the ranges of the prior distribution.

**Figure 5:**
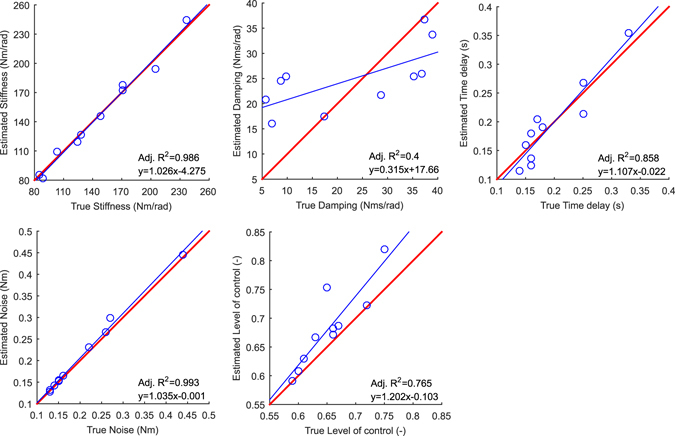
Estimated parameters (posterior mean values) against true parameters. The equation for the estimated parameters against the true parameters is presented with a blue thin line. The equation should ideally be *y* = *x*, as indicated with a red thick line. The corresponding adjusted *R* ^*2*^ values are shown in the figures.

To further investigate accuracy of the inference, we calculated the posterior mean of the parameter values. The true parameter values are presented in Section Methods (Test subjects and measurements). The posterior mean values (±SD) for the ten simulated subjects were: *P* = 146 ± 52 Nm/rad, *D* = 25 ± 7 Nms/rad, *Δ* = 0.19 ± 0.07 s, *σ* = 0.21 ± 0.10 Nm, *C*_*ON*_ = 0.68 ± 0.07. Figure 4 presents an example of marginal PDFs for the five parameters and for one simulated test subject (the one presented in the rightmost panel in Fig. 3). Figure 5 shows the true parameter values against the inferred posterior mean values for all ten simulated subjects. These figures show high correlation between all five true and inferred parameter sets, except for parameter *D*. Furthermore, the average error between the true and inferred parameter values, [100% * (Inferred parameter − True parameter)/True parameter], confirmed that we were able to infer *P*, *Δ*, *σ*, and *C*_*ON*_ parameters accurately, but also that we were unable to infer *D*: *P*_*error*_ = −1 ± 4%, *D*_*error*_ = 63 ± 109%, *Δ*_*error*_ = −1 ± 15%, *σ*_*error*_ = 3 ± 3%, and *C*_*ON,error*_ = 4 ± 5%. However, the higher error observed for *D* nevertheless had only a minor impact on the predictive accuracy of the model considering that the original COM signal summary statistics falls within the 95% CIs of the inferred summary statistics (Fig. 3).

**Figure 6:**
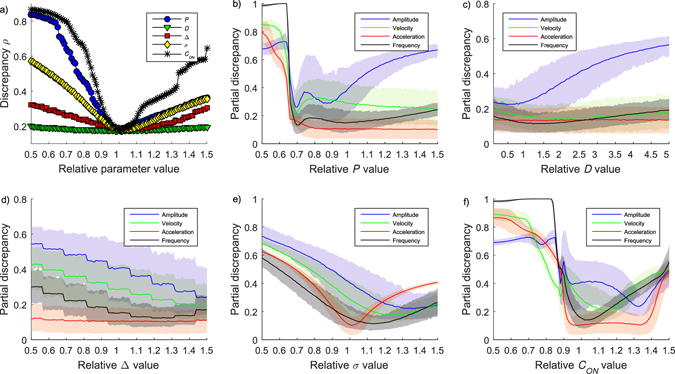
Sensitivity analysis. (**a**) The results are averaged (mean discrepancy and 95% CIs) across the 10 simulated subjects and 100 simulation rounds per subject. All summary statistics are included. (**b**–**f**) Amplitude-, velocity -, acceleration- histograms, and spectrum used one at the time to form the summary statistics. The results are averaged across 1000 simulation rounds of one representative test subject, the subject presented in the rightmost panel in Fig. 3. and in Fig. 4. The parameters are (**b**) stiffness, *P*, (**c**) damping, *D* (please note the wider x-axis scale, from 0.1 to 5), (**d**) time delay, *Δ*, (**e**) noise, *σ*, and (**f**) level of control, *C*_*ON*_. Briefly, the steeper the curve the more effectively the summary statistics detects changes in model parameters.

Figure 6 shows the results of the sensitivity analysis. The most influential parameters, *C*_*ON*_, *P*, and *σ* were the most accurately inferred, while the least influential parameter *D* was also the hardest one to infer. To see an increase in the discrepancy, the value of *D* needed to be changed more than rest of the parameters –between 0.1 and 5 times of its real value (Fig. 6c). In (Fig. 6b–f), the spectrum and the histogram of the amplitude, the velocity, or the acceleration is each used one at a time to calculate the summary statistics (see Section Methods: Statistical inference of the model parameters). This analysis shows relations between summary statistics and model parameters: A change in *P* was detected by all summary statistics, though most clearly as a change in COM amplitude. Especially, too low a *P* value lead to a rather sudden and significant increase in discrepancy, *ρ* (Eq. (10)). A change in *D* leads to only a minor change mostly in COM amplitude and frequency, while a change in *Δ* was most clearly seen in COM frequency. A change in *σ* was detected by all summary statistics, but most clearly in acceleration information. Finally, a change in *C*_*ON*_ was visible in all of the summary statistics.

To understand the relative effects of *P* and *D* on the model output, we studied the relative effect of *P* on corrective torque *T*_*C*_, *f*_*P*_(*θ*(*t* − *Δ*)), compared to that of *D*, *f*_*D*_($\dot{\theta }$ (*t* − *Δ*)), Eq. (2). The effect of *P* is ca. 50-times larger than the effect of *D*, when the default values of the sway model^21^ are used (see Section Methods: The control model). Even when the value of *D* was increased to 100 Nms/rad, the effect of *P* is still ca. 3-times larger than that of *D* on the corrective torque.

### Real subjects

**Figure 7:**
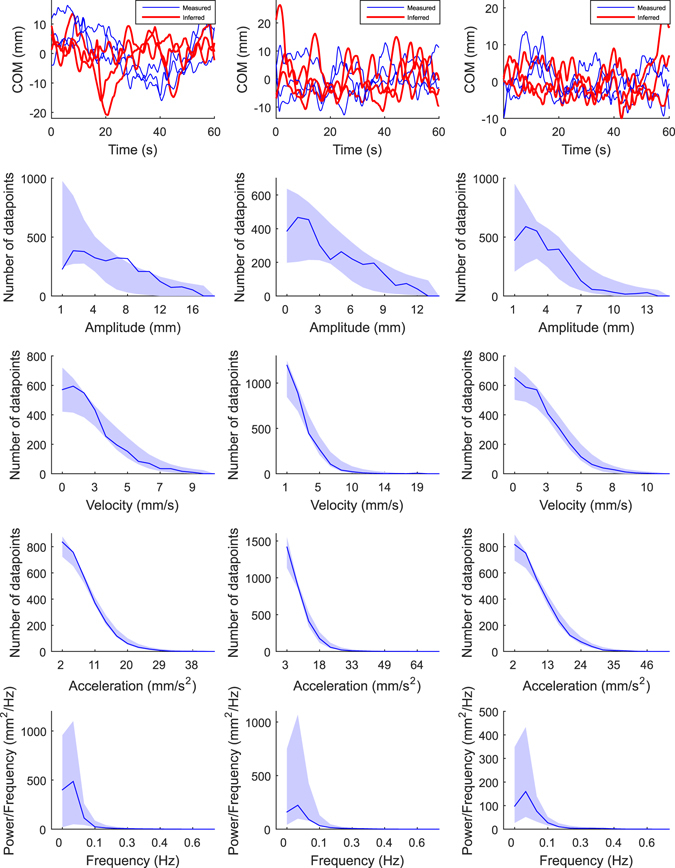
Real COM sway signals (top panel) and corresponding summary statistics (lower panels). The three columns present three real subjects. The blue COM curves correspond to the measured signals. The red COM signals represent values simulated using parameters that were sampled from the joint posterior PDFs that were inferred from the measured COM signals by the SMC-ABC algorithm. The lower panels show the summary statistics: amplitude -, velocity -, and acceleration histograms and spectra (see Section Methods: Statistical inference of the model parameters). In each figure, the blue line is the true summary statistic calculated from the original COM signals, and the blue shadowed regions present 95% CIs that were calculated using the COM signals that were simulated using parameters that were sampled from the inferred marginal posterior PDFs. The mass and estimated height of COM (see Section Methods: The control model) are: leftmost column: *m* = 68 kg and *h* = 0.80 m; mid column: *m* = 66 kg and *h* = 0.89 m; rightmost column: *m* = 68 kg and *h* = 0.87 m.

**Figure 8:**
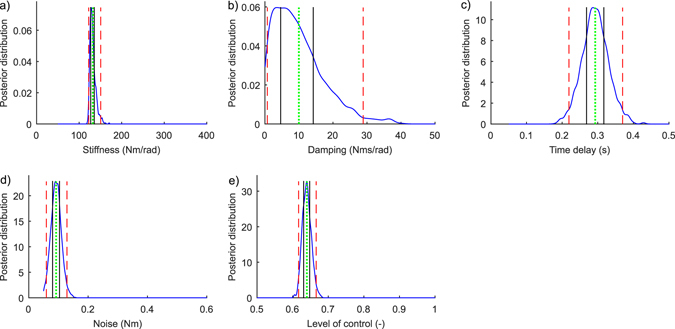
Marginal posterior probability density functions for the five parameters (real subject, same one as in Fig. 7, mid panel): (**a**) Stiffness, *P*; (**b**) Damping, *D*; (**c**) Time delay, *Δ*; (**d**) Noise, *σ*; and (**e**) Level of control, *C*_*ON*_. Vertical lines present estimated parameter values (green, dotted), 50% CIs (black, solid), and 95% CIs (red, dashed). The ranges on the x-axes correspond to the ranges of the prior distribution.

Figure 7 presents the measured and inferred COM signal from three different real subjects. As was the case with simulated and inferred COM signals (Fig. 3) the inferred COM signals are difficult to distinguish from the measured COM signals by eye. The lower panels in Fig. 7 show the summary statistics that are used to compare the original COM signals and the inferred COM signals. The summary statistics calculated from the measured COM signals fit into the 95% CI area of the summary statistics that describes the COM signals that were simulated using the inferred parameters. Figure 8 presents an example of marginal PDFs for the five parameters and for one real subject (same subject as in the mid panel in Fig. 7). The posterior mean (±SD) values for the 10 real subjects were: *P* = 156 ± 55 Nm/rad, *D* = 16 ± 6 Nms/rad, *Δ* = 0.30 ± 0.02 s, *σ* = 0.12 ± 0.04 Nm, *C*_*ON*_ = 0.61 ± 0.04.

Since the true parameter values of the real subjects are unknown, we compared sway measures (Eqs (11)–(14) and Section Methods: Sway measures) that were calculated using both the measured and inferred COM signals. Separate paired *t*-tests between the measured COM signals (real subjects) and the COM signals that were simulated using the inferred parameter values showed significant difference between mean acceleration (*MA*) values (*p* < 0.01), but not between mean distance (*MD*), mean velocity (*MV*), mean frequency (*MF*), fuzzy sample entropy (*FSE*), scaling exponent (*α*), correlation dimension (*D*_*2*_), and largest Lyapunov exponent (*λ*_*max*_) values (Table 1). For the latter seven summary statistics the predictive distribution is centered close to the summary statistics calculated from the real data.

**Table 1:** Predictive accuracy of the inference with real subjects.

	True	Inferred	Sig.
*MD* (mm)	4.6 ± 1.6	4.1 ± 1.1	NS.
*MV* (mm/s)	2.7 ± 0.4	2.7 ± 0.5	NS.
*MA* (mm/s^2^)	8.9 ± 1.8	9.8 ± 2.3	*p* < 0.01
*MF* (Hz)	0.11 ± 0.03	0.12 ± 0.03	NS.
*FSE*	0.12 ± 0.01	0.13 ± 0.02	NS.
*α*	1.7 ± 0.1	1.7 ± 0.1	NS.
*D*_*2*_	2.3 ± 0.5	2.1 ± 0.5	NS.
*λ*_*max*_	0.59 ± 0.15	0.61 ± 0.16	NS.

Basic sway measures (Eqs (11–(14)) and nonlinear sway measures (Section Methods: Sway measures) calculated from real COMs (“True”) and the inferred COMs (“Inferred”, e.g. calculated from COM signals that were simulated with the inferred parameters). “NS.” means not significant. These results show that the real COMs and the inferred COMs are similar, except for their mean acceleration values.

## Discussion

This study was conducted to determine whether a SLIPM model with intermittent control together with approximate Bayesian computation can infer sway signals and parameters that are plausible for human subjects. Reliable inference could thereby result in better understanding of how different physiological conditions alter the way balance is maintained.

The performance of the ABC inference approach was quantified for simulated test subjects by calculating the fractional error (see Section Methods: Statistics) and the goodness of fit (adjusted R^2^) between true and estimated parameters. Calculating the error between the true and inferred parameter values showed that even though the error between *P*, *Δ*, *σ*, and *C*_*ON*_ on average was less than 5% (standard deviation at most 15%), the error in *D* inference was large, *D*_*error*_ = 63 ± 109%. These results indicate that in case of *C*_*ON*_, there might be a small bias toward a larger value, which is of negligible practical concern. Our results show that our summary statistics did not permit accurate inference of *D*. However, this did not adversely affect the predictive ability of the inferred model. Fitting the estimated parameter values against the true parameter values confirmed the results with fractional errors: the adjusted *R* ^*2*^ value for *D* was only 0.400, while it was 0.765–0.993 with the other parameters (Fig. 5). Consequently, it appears that the SMC-ABC inference method together with the chosen summary statistics capture the main features of the simulated COM signals.

Figure 6 presents the results of the sensitivity analysis. While the model contains many parameters, it can well be that some of them have a more significant effect on the postural sway than others. (For example, consider a model for a ball flying in (thin) air –although the dynamics includes a drag force, in many cases the effect of the drag is not very significant compared to other effects, as measurements would indicate.) Indeed, our study suggests that not all model parameters are equally influential on the model output: those parameters that were most easily inferable (*P*, *σ*, and also *C*_*ON*_) had generally most influence on the model output. Importantly, changing the *D* value between 0.5 to 1.5 times of its true value changed the model output only marginally as compared to the other model parameters. It is important to note that the sensitivity analysis we performed contained the net result of several parts of our method: stochastic variance that depends on e.g. chosen signal length, the chosen summary statistics, and the chosen discrepancy value – but not on the optimization part of SMC-ABC.

To further understand the difficulty to infer the *D* parameter, we compared the relative effects of *P* and *D* on the model output. These two parameters are similar in the sense that they are both used to maintain the pendulum in an upright stance via corrective torque, *T*_*C*_. Since the *θ* signal is relatively smooth (with 50 Hz sampling frequency), the magnitude of $\dot{\theta }$ is smaller than that of *θ*. Also, the magnitude of *D* is smaller than that of *P*. Consequently, the effect of *P* on the corrective torque is ca. 50-times larger than the effect of *D* with parameter default values (see Section Methods: The control model). Even when the value of *D* was increased to 100 Nms/rad, the effect of *P* is still ca. 3-times larger than that of *D*. Therefore, the effect of *D* – that is weaker yet similar to the effect of *P* – may go unnoticed. Again, it is important to note, that this dominance of *P* over *D* is inherent to the sway model. Hence, the easiest and perhaps only way to substantially increase the accuracy of inferring *D* is to increase the simulation length which decreases the variance of the summary statistics and the discrepancy value. This may, however, not be a viable option since it increases the duration of the posturographic measurements beyond reason. Considering both the results of our sensitivity analysis and the intrinsic dominance of *P* over *D*, the difficulty to accurately infer *D* may not be surprising.

Since no true parameter values are available for the COM signals for real subjects, the goodness of fit was estimated by investigating the differences between sway measures calculated from the real COMs and those calculated from COMs simulated using the inferred parameters. We found that the mean acceleration of the simulated COM signals exceeded that of the measured COM signals (*p* < 0.001). The reason for the discrepancy regarding the mean acceleration may be that the expected value of this quantity is not a smooth enough function of the model parameters. Differences in measured and simulated signals may also be due to reasons related to the sway model: First, an accurate replication of nonstationarities in body sway, e.g. voluntary movements or small alterations in stance, is challenging. Second, the musculoskeletal model is a simplification of the kinetics of the human body; SLIPM presumes only one link, the ankle, to be engaged in the sway. Third, the Asai *et al*. model was constructed using a 60 kg subject with COM height of 1 m, and *I* = 60 kgm^2^. Our subjects exhibited great inter-individual differences in anthropometrics, which may lead to difficulties in applying (extrapolating) the model. However, most sway measures (*MD*, *MV*, *MF*, *FSE*, *α*, *D*_*2*_, *λ*_*max*_) showed no difference between measured and simulated COM signals. Consequently, it appears that the simulations and inference capture the main features of the body sway for most subjects.

Future work should focus on choosing an even faster inference method, e.g. Bayesian optimization for likelihood free inference (BOLFI), that was presented by Gutmann and Corander 2016^40^. Further exploration of summary statistics could help resolve whether the active damping, *D*, can be inferred from COM data, and if so, find measures that more accurately infer *D*.

## Methods

All signal processing was done in Matlab (R2015a, The MathWorks, Inc., USA). All AP signals were recorded using *f*_*S*_ = 50 Hz sampling frequency, and set to zero-mean.

### The control model

Figure 1 in the Results Section presents the schematic of the sway model. The sway of an upright standing human can be modelled as a single-link inverted pendulum^21^:

1 $$I\ddot{\theta }(t)={T}_{tot}={T}_{g}(t)-{T}_{c}(t)+{T}_{d}(t).$$

Here *I* is the moment of inertia of the body (appr. *mh* ^*2*^), $\ddot{\theta }$ is the second derivative with respect to time *t* of the tilt angle, *θ*, *T*_*g*_ is the gravitational torque, *T*_*d*_ is the disturbance torque (sensory noise, pulse, hemodynamics), and *T*_*c*_ is the corrective ankle torque that counteracts *T*_*g*_ and *T*_*d*_. By inserting *T*_*g*_, *T*_*c*_, and *T*_*d*_ into Eq. (1)^21^, this equation becomes a stochastic delay differential equation (SDDE)^41, 42^:

2 $$I\ddot{\theta }(t)=mgh\theta (t)-[K\theta (t)+B\dot{\theta }(t)+{f}_{P}(\theta (t-{\rm{\Delta }}))+{f}_{D}(\dot{\theta }(t-{\rm{\Delta }}))]+\sigma \xi (t),$$

where *m* is the body mass, *g* = 9.81 m/s^2^, *h* is the distance between 3D center-of-mass and the ankle joint (appr. *h* = 0.55 ∙ *h*_*sub*_ − *h*_*F*_, where *h*_*sub*_ is the subject’s height and *h*_*F*_ = 0.085 m is the coordinate of the ankle joint), and *ξ* is Gaussian noise (zero mean and unit variance). The active and passive PD-controllers’ gain parameters relate to the stiffness (*P* and *K*), and damping (*D* and *B*) of the motion of the modelled subject. The time delay, *Δ*, describes time used to conduct and integrate neural signals, and to initiate muscle contraction. The noise intensity, *σ*, represents the subject’s internal perturbations. ${f}_{P}(\theta (t-{\rm{\Delta }}))$ and ${f}_{D}(\dot{\theta }(t-{\rm{\Delta }}))$ are active stiffness and active damping. The active stiffness and damping are ‘ON’ intermittently (model 4 in Asai *et al*.^21^) (Fig. 1, upper left corner):

3a $$\{\begin{matrix}\,{f}_{P}(\theta (t-{\rm{\Delta }}))=P\theta (t-{\rm{\Delta }})\\ \,{f}_{D}(\dot{\theta }(t-{\rm{\Delta }}))=D\dot{\theta }(t-{\rm{\Delta }})\end{matrix}$$

if $\theta (t-{\rm{\Delta }})(\dot{\theta }(t-{\rm{\Delta }})-{a}_{s}\theta (t-{\rm{\Delta }})) > 0$, and ${\theta }^{2}(t-{\rm{\Delta }})+{\dot{\theta }}^{2}(t-{\rm{\Delta }}) > {r}^{2}$, and.

3b $$\{\begin{matrix}\,{f}_{P}(\theta (t-{\rm{\Delta }}))=0\\ \,{f}_{D}(\dot{\theta }(t-{\rm{\Delta }}))=0\end{matrix}$$

otherwise. The radius *r* determines a quiet zone (active control is ‘OFF’) around the equilibrium point (upright stance). The slope *a*_*s*_ determines the level of control, *C*_*ON*_, (i.e. fraction of active torqueing in the phase plane, AREA_ON_/(AREA_ON_ + AREA_OFF_)), according to *C*_*ON*_ = 0.5 + atan(−*a*_*s*_)/π (Fig. 1A). The default values for the parameters from Asai *et al*.^21^ are *K* = 0.8 · *mgh* Nm/rad, *B* = 4 Nms/rad, *P* = 0.25 · *mgh* Nm/rad, *D* = 10 Nms/rad, *Δ* = 0.2 s, *σ* = 0.2 Nm, *r* = 0.004 rad-rad/s, and *C*_*ON*_ = 0.62 (*a*_*s*_ = −0.4).

We first analyze the case when the active control is ‘ON’. By rearranging the terms in Eq. (2), and by transforming the variables into a more convenient form (*a* = (*mgh* − *K*)/*I*, *b* = −*B*/*I*, *c* = −*P*/*I*, *d* = −*D*/*I*, and *e* = *σ*/*I*), Eq. (2) becomes:

4 $$\ddot{\theta }(t)=a\theta (t)+b\dot{\theta }(t)+c\theta (t-{\rm{\Delta }})+d\dot{\theta }(t-{\rm{\Delta }})+e\xi (t).$$

By converting the second order SDDE into two first order SDDEs one obtains:

5 $$\{\begin{matrix}\,\dot{\theta }(t)=\omega (t)\\ \,\dot{\omega }(t)=a\theta (t)+b\omega (t)+c\theta (t-\Delta )+d\omega (t-\Delta )+e\xi (t),\end{matrix}$$

where *ω* is angular velocity. Next, discretizing the pair of equations using the Euler-Maruyama method^41, 42^ leads to:

6 $$\{\begin{matrix}\,{\Theta }_{n+1}={\Theta }_{n}+{\Omega }_{n}\Delta t\\ \,{\Omega }_{n+1}={\Omega }_{n}+(a{\Theta }_{n}+b{\Omega }_{n}+c{\Theta }_{n-k}+d{\Omega }_{n-k})\Delta t+e{\xi }_{n}\sqrt{\Delta t}\end{matrix}$$

Here *Θ* and *Ω* are the discretized sway angle and angular velocity, *Δt* is the time increment (sampling interval, in seconds), *n* is time index, whereas *k* = *Δ*/*Δt* is unitless time delay, *ξ*_*n*_ are independent normally distributed random numbers with zero mean and unit variance. When the active control is ‘OFF’, *c* = *d* = 0, and Eqs (4)–(6) simplify accordingly.

The initial angle and angular velocity:

7 $$\{\begin{matrix}{{\rm{\Theta }}}_{0} & = & \eta \\ {{\rm{\Theta }}}_{-k < n < 0} & = & {{\rm{\Omega }}}_{-k < n < 0}|=0,\end{matrix}$$

where *η* is a uniformly distributed random number between −0.01 and 0.01 radians. The pendulum height *h* and weight *m* were chosen according to the subject’s height and weight. The passive stiffness and damping were constant and chosen according to Asai *et al*.^21^: *K* = 0.8 · *mgh* Nm/rad and *B* = 4.0 Nms/rad. Five model parameters (*P, D*, *Δ*, *σ*, and *C*_*ON*_) were chosen for optimization.

The transformation from *θ* to COM is:

8 $$COM(t)=h\,\sin (\theta (t)).$$

To compare the measured COP signal with the simulated COM two approaches are possible: either one converts the simulated COM to COP, which requires numerical differentiation of COM, or one converts the measured COP to COM. To avoid complications associated with numerical differentiation of noisy signals we employed the latter approach using the Laplace transform^5^:

9 $$CO{M}_{n}=\frac{{\rm{\Delta }}t\sqrt{\frac{g}{h}}}{2}{e}^{-|n{\rm{\Delta }}t|\sqrt{\frac{g}{h}}}\ast CO{P}_{n},$$

where ^*^ denotes convolution. The algorithm to implement Eq. (9) is presented by Tossavainen 2006^6^.

### Test subjects and measurements

The employed protocol was accepted by the ethical review board of the University of Helsinki, and conducted in accordance with the Declaration of Helsinki. Written informed consent was obtained prior to the tests from each subject.

Our cohort comprised 10 subjects (4 males and 6 females, 29 ± 5 years, 68 ± 16 kg, 170 ± 13 cm, BMI 23 ± 3 kg/m^2^, no medication or diagnose affecting balance). Their COP signals were recorded with a Nintendo Wii Fit balance board^43^. The subjects stood erect, feet together and hands folded across their chest, looking at a marker 70 cm in front of them on a wall. Each measurement comprised three repeats of 60-second trials with a 30 s pause between each trial. The measurement program –a custom made C# program that uses an open source WiiMoteLib-library^44^ – was run on a PC laptop with Bluetooth^®^ access to the Wii board.

To test the accuracy of the inference, we further simulated 10 test subjects (66 ± 17 kg, 169 ± 12 cm, BMI 23 ± 6 kg/m^2^, *P* = 146 ± 50 Nm/rad, *D* = 23 ± 14 Nms/rad, *Δ* = 0.20 ± 0.06 s, *σ* = 0.21 ± 0.10 Nm, and *C*_*ON*_ = 0.65 ± 0.05). Height, weight and model parameters were randomly varied, and any test subject that met the following criteria were accepted: the maximum sway amplitude was between 10 mm and 50 mm, height was between 145 cm and 200 cm, weight was between 40 kg and 110 kg, BMI was between 15 and 35, and the sway looked realistic when examined visually.

### Statistical inference of the model parameters

For Bayesian inference, we needed to specify the prior distribution of the parameters. We assumed that they are statistically independent and uniformly distributed on the following intervals: [50, 400] Nm/rad for *P*; [0.05, 50] Nms/rad for *D*; [0.05, 0.5] s for *Δ*, [0.05, 0.6] Nm for *σ*, and [50, 100] for *C*_*ON*_. The model in our paper is too complex for the likelihood function to be calculated analytically in closed form, or to be calculated numerically with high accuracy. This prevented us from using standard likelihood-based inference methods. Since we can simulate data from the model for any values of the parameters, statistical inference can be performed using the approximate Bayesian computation (ABC) approach for intractable simulator-based models.

In a nutshell, ABC approximates the likelihood function using a discrepancy function that measures the similarity between the observed and the simulated data. Parameter values are assigned a large likelihood if they are very probable to generate data similar to the observed data. Small likelihoods are assigned if the probability to generate similar data is very small. For further information on ABC, we refer the reader to a review paper by Lintusaari *et al*.^36^.

We followed the common practice in ABC to summarize each data set by lower dimensional summary statistics, and to compute the discrepancy based on the summary statistics rather than the full data^45^. Studying the sway model (Fig. 1 and Eq. (2)) may give an indication on how to choose the summary statistics. Equation (2) contains sway angle *θ*, angular velocity $\dot{\theta }$, and angular acceleration, $\ddot{\theta }$ that are closely related to the COM signal (Eq. (8)), its velocity and acceleration. Stiffness, *P*, and damping, *D* are gain parameters that both are part of the corrective torque, *T*_*c*_, that keeps the pendulum upright. Stiffness relates directly to the magnitude of the *θ* signal (Eq. (3a)): the larger the sway amplitude, the larger the corrective movement due to stiffness. Damping relates to the time derivative of *θ*, $\dot{\theta }$ (Eq. (3a)); the larger the $\dot{\theta }$, the larger the effect of damping on *T*_*c*_. The level of control, *C*_*ON*_, governs the damping and stiffness parameters, it determines in practice when *PD-*control is ON. The time delay, *Δ*, impairs the actions of the PD-control by delaying it. Further, *σ*, is the intensity of the Gaussian noise in the disturbance torque, *T*_*d*_. The parameters *P*, *D*, *C*_*ON*_, and *Δ* drive the acceleration $\ddot{\theta }$ by coupling to *θ* and $\dot{\theta }$ terms, and characterize the dynamics of the pendulum system (Eq. (2)). Therefore, we anticipate the effect of these parameters to be visible in all three *θ*, $\dot{\theta }$, and $\ddot{\theta }$ signals. In contrast, the Gaussian noise term parameterized by the intensity *σ* appears as a driving force, affecting the acceleration $\ddot{\theta }$ directly (Eq. (2)). Therefore, we anticipate the effect of *σ* to be most visible in the $\ddot{\theta }$ signal. Because of these reasons, changes in the model parameters should be visible in the COM signal, as well as in its velocity -, acceleration -, and frequency transforms.

Denoting the vector of summary statistics of the observed and simulated data by *Ф*_*obs*_ and *Ф*_*sim*_, respectively, the discrepancy *ρ* was computed as the normalized relative error between them:

10 $$\rho =\frac{1}{l}\sum _{i=1}^{l}\frac{|{{\rm{\Phi }}}_{obs}-{{\rm{\Phi }}}_{sim}|}{|{{\rm{\Phi }}}_{obs}+{{\rm{\Phi }}}_{sim}|},$$

where *l* = 60 is the length of the summary statistics. Let us denote COM signals that are transformed from the measured COP signals according to Eq. (9) ‘measured COM signals’. Summary statistics *Ф*_*obs*_ and *Ф*_*sim*_ were calculated from both simulated and measured COM signals that were first filtered with a bidirectional FIR filter with 10 Hz lowpass cutoff frequency. The absolute value of the COM amplitude, |*x*|, velocity, *f*_*S*_ ∙ |*x*(*i* + 1) − *x*(*i*)|, and acceleration, *f*_s_^2^ ∙ |[*x*(*i* + 2) − *x*(*i* + 1)] − [*x*(*i* + 1) − *x*(*i*)]|, were represented as histograms with 15 bins each. Bin boundaries were individually chosen for each subject according to the maximum amplitude, velocity, and acceleration values of the three measured COM signals. Matlab’s function ‘pwelch’ was used on the COM signal to calculate the power spectral density (PSD). The PSD vector up to 0.7 Hz featured 15 data points and had therefore the same weight (importance) as the amplitude, velocity, and acceleration histograms each having 15 bins. The bins/PDS values of the three repeated 60 s COM trials were averaged. This kind of vector comprising 60 data points was taken as summary statistics to describe each of the data sets.

We used a sequential (population) Monte Carlo implementation of approximate Bayesian computation (SMC-ABC)^46^. In each iteration, the algorithm ran the sway simulation with different candidate parameter values, calculated the summary statistics *Ф*_*obs*_ and *Ф*_*sim*_, and determined the discrepancy *ρ* between the observed and simulated data set until a preset number of simulations produced discrepancies that were equal or smaller than a threshold *ε*. The corresponding “accepted” parameter values can be shown to be samples from an approximation of the posterior distribution of the parameters given the observed data. The point of the SMC-ABC algorithm is that the threshold *ε* is made smaller in each iteration, which makes the approximation more accurate. In the algorithm the candidate parameter values are determined in an adaptive manner based on the samples obtained in the previous iteration. In the first iteration, the parameter values are drawn from the prior. We ran the SMC-ABC algorithm for seven iterations, accepting 5000 samples per iteration. For further information on SMC-ABC, see e.g. the review by Lintusaari *et al*.^36^.

In our case the outcome of the SMC-ABC algorithm are samples from the joint posterior probability density function of the five parameters we investigate. Here, we used posterior mean values of each marginal PDF to summarize information about that PDF. The posterior mean values are compared to the original parameter values to estimate the goodness of the inference (e.g. Fig. 5), and to summarize the results from all ten simulated and ten real subjects.

### Sway measures

To test the quality of the inferred COM signals, we calculated four conventional statistical sway measures from the measured COM signals and their inferred counterparts: mean distance *MD*, mean velocity *MV*, mean acceleration *MA*, and mean frequency *MF* ^7^.

11 $$MD=\frac{1}{n}\sum _{i=1}^{n}|x(i)|$$

12 $$MV=\frac{1}{n-1}\sum _{i=1}^{n-1}|[x(i+1)-x(i)]\cdot {f}_{S}|$$

13 $$MA=\frac{1}{n-2}\sum _{i=1}^{n-2}|[x(i+2)-x(i+1)]-[x(i+1)-x(i)]\cdot {f}_{S}^{2}|$$

14 $$MF=\frac{MV}{2\pi \cdot MD},$$

where *x* is the COM signal of length *n*, and *f*_*S*_ = 50 Hz is the sampling frequency. When calculated from the COP signals, these simple sway measures correlate only moderately with each other^9^. This means that they possibly represent different aspects of the body sway.

To get a detailed picture of the characterization of body sway, we additionally used four nonlinear sway measures: fuzzy sample entropy (FSE)^47^, scaling exponent *α* of detrended fluctuation analysis (DFA)^48^, correlation dimension (*D*_*2*_)^49, 50^, and largest Lyapunov exponent (*λ*_*max*_)^51, 52^. These measures have been used to characterize body sway, and one can theorize that they relate to physiological aspects of human body^50^. Commonly these measures are used to characterize center-of-pressure (COP) signals, here we use them to characterize the COM signals – the model output.

Fuzzy sample entropy^47^ describes the repeatability or predictability of the signal. Its change in sway signals has been theorized to signify the amount of attention a person invests in balancing^53^. The algorithm calculates the distance *d* ^*L*^_*i*,*j*_ = max_0≤*k*≤*L−1*_|*x*_*j+k*_ − *x*_*i+k*_| between all *L*-length sequences *x*_*i*_ and *x*_*j*_ (*i* ≠ *j*) in the signal. Next, a (fuzzy) value between 0 and 1 is determined using the function *μ*(*d* ^*L*^_*i*,*j*_,*r*,*c*) = exp(−(*d* ^ln(*c*ln2)/ln*r*^)/*c*); the more alike the sequences are, the larger the *μ* value. The tolerance *r* and the shape factor *c* determine the shape of *μ*. *B*_*m*_ is the sum of *μ*-values for all combinations of *x*_*i*_ and *x*_*j*_. The procedure is repeated with *L* + 1-length sequences (*d* ^*L*^_*i*,*j*_ = max_0≤*k*≤*L*_|*x*_*j*+*k*_ − *x*_*i*+*k*_|) which gives *A*_*L*_, as the sum of the *μ*-values. Finally, FSE is defined as −ln(*A*_*L*_/*B*_*L*_). We chose the *L* and *r* parameters as instructed by Lake *et al*.^54^: The segment length *L* was chosen according to the corresponding AR-process order, which was determined by minimizing the Schwarz’s Bayesian criterion. *r* was chosen based on minimizing a sample entropy (FSE, except that *μ* is a Heaviside function) error estimate. Using this approach, we arrived at: *L* = 6, *r* = 0.03 and *c* = 0.01.

Detrended fluctuation analysis^48^ quantifies long-range correlations in nonstationary signals. The algorithm first numerically integrates the signal. The signal is then divided into *s*-length segments (here we chose *s* to be between 5 and 750 data points with logarithmic intervals), and each segment is separately detrended by a linear least squares fit. The square root of the average residuals of the segments is plotted on a logarithmic scale against the segment length *s*. The scaling exponent *α* is the slope of the so constructed graph. *α* is between 0 and 2. The larger the value, the more persistent, ‘smoother’ the signal is^48, 55^.

Correlation dimension estimates the number of the active control variables (degrees of freedom) of the underlying dynamics of postural control^49, 50^. The COM signal *x* is presented in a state-phase presentation, *X*_*i*_ = [*x*_*i*_, *x*_*i+J*_, *x*_*i+2J*_, …, *x*_*i+(M-1)J*_], where *J* is the lag, *M* the embedding dimension, and *X*_*i*_ a point in a *N* − (*M* − 1)*J* length trajectory. Here *J* was estimated as the first minimum of the mutual information function^56^. The algorithm calculates the correlation sum *C*_*M*_, the fraction of pairs of trajectory points that are separated by a distance less than *r*, but by more than the temporal separation of twice the lag *J*. *C*_*M*_ behaves as a power law, *C*_*M*_(*r*) ∝ *r* ^*D2*^ for small values of *r*. *d*_*M*_ is the slope of *C*_*M*_ against *r* on logarithmic scale. *d*_*M*_ is calculated for increasing *M* (*M* = 1, 2, 3…). When *M* > 2 ∗ *d*_*M*_ + 1, *d*_*M*_ is the correlation dimension, *D*_*2*_. Here *M* = 6 ± 1 and *J* = 27 ± 5.

A positive value of the largest Lyapunov exponent indicates the presence of chaos in a deterministic and nonlinear signal^51, 52^. When applied to COM signals, *λ*_*max*_ quantifies the sensitivity of the postural control system to small, internal perturbations^57^. The *λ*_*max*_ algorithm uses the state-phase presentation of the signal, as described in the previous paragraph on *D*_*2*_. Next, the divergence of initially close trajectories is quantified using nearest neighbours, *X*_*ĵ*_, where *X*_*ĵ*_ is defined as the *X*_*j*_ that minimizes the Euclidean distance between *X*_*j*_ and *X*_*ĵ*_, and still has a temporal separation greater than twice the lag *J*. The divergence at an instance *i* is *d*(*i*)_*j*_ = *d*(0)_*j*_e^*λmax*(*iΔt*)^, where *d*(0)_*j*_ is the initial distance between *X*_*j*_ and *X*_*ĵ*_. Finally, *λ*_*max*_ is estimated as the slope of a least-squares fitted line, *y*(*i*) = *Δt* ^−1^〈ln*d*(*i*)_*j*_〉, where 〈 〉 is the average value over *j* neighbours.

### Statistics

The inference error was estimated from the results of both the simulated and the real subjects. The error between the true parameters and inferred parameters (simulated subjects) was estimated by regressing the estimated parameters (posterior mean) against the true parameters, and by calculating the fractional error: 100% * (Estimated parameter-True parameter)/True parameter. Separate paired *t*-tests were conducted to estimate the difference between the sway measures calculated from the *measured* COMs and the COMs that were simulated with the inferred parameters. We considered *p* < 0.05 to be significant.

To understand how changes in parameters mediate to changes in summary statistics, we analyzed the sensitivity of each parameter. We changed one parameter value at time between 0.5 and 1.5 times its true value (between 0.1 and 5 in case of *D*), while keeping the others fixed, and calculated the discrepancy value, *ρ* (Eq. (10)). We also formed the summary statistics, *Ф*_*sim*_ and *Ф*_*obs*_ using either the amplitude -, the velocity -, the acceleration histogram or the spectrum (see Section Methods: Statistical inference of the model parameters). We then repeated the sensitivity analysis using these four summary statistics. Furthermore, we compared the relative effects of *P* and *D* (Eq. 3) on the model output. Both *P* and *D* affect how the pendulum is maintained upright by the corrective torque, *T*_*C*_. To this end, we simulated 6000 s of upright stance using default parameter values (*P* = 147 Nm/rad, *D* = 10 Nms/rad, *K* = 471 Nm/rad, *B* = 4 Nms/rad, *σ* = 0.2 Nm, *Δ* = 0.2 s, *r* = 0.004 rad-rad/s, *m* = 60, *h* = 1), and another 6000 s where all parameters had their default values, except *D* = 100 Nms/rad (10 times its default value).
